# Gate Control Mechanisms of Autoencoders for EEG Signal Reconstruction

**DOI:** 10.3390/s25113389

**Published:** 2025-05-28

**Authors:** Kangjing Li, Heba El-Fiqi, Min Wang

**Affiliations:** 1 School of Systems and Computing, University of New South Wales, Canberra, ACT 2612, Australia; kangjing.li@canberra.edu.au (K.L.); h.el-fiqi@unsw.edu.au (H.E.-F.); 2 Faculty of Science and Technology, University of Canberra, Canberra, ACT 2617, Australia

**Keywords:** autoencoder (AE), gate control, data reconstruction, neural networks, unsupervised learning

## Abstract

Electroencephalography (EEG) is a non-invasive and portable way to capture neurophysiological activity, which provides the basis for brain–computer interface systems and more innovative applications, from entertainment to security. However, the acquisition of EEG signals often suffers from noise contamination and even signal interruption problems due to poor contact of the electrodes, body movement, or heavy noise. Such heavily contaminated and lost signal segments are usually removed manually, which can hinder practical system deployment and application performance, especially in scenarios where continuous signals are required. In our previous work, we proposed the weighted gate layer autoencoder (WGLAE) and demonstrated its effectiveness in learning dependencies in EEG time series and encoding relationships among EEG channels. The WGLAE adopts a gate layer to encourage the AE to approximate multiple relationships simultaneously by controlling the data flow of each input variable. However, it only applies a sequential control scheme without taking into account the physical meaning of EEG channel locations. In this study, we investigate the gating mechanism for WGLAE and validate the importance of having a proper gating scheme for learning relationships between EEG channels. To this end, several gate control mechanisms are designed that embed EEG channel locations and their corresponding underlying physical meanings. The influences introduced by the proposed gate control mechanisms are examined on an open dataset with different scales and associated with various stimuli. The experimental results suggest that the gating mechanisms have varying influences on reconstructing EEG signals.

## 1. Introduction

Understanding brain activity patterns is fundamental to unravelling the mysteries of cognitive processes, neurological disorders, and brain-related phenomena. Electroencephalography (EEG) has emerged as a vital tool in the field of neuroscience and brain–computer interfaces, offering a non-invasive means to study and exploit the electrical activity of the brain [1,2]. However, the acquisition of EEG signals is often impeded by various challenges, including noise, artefacts, and missing channels, which cause signal discontinuity and contamination [1,3]. The reconstruction of EEG signals has thus become a critical task, as it aims to enhance the quality and reliability of recorded brain activity data. Enhanced EEG data quality and quantity facilitate more precise analysis, leading to a deeper comprehension of neural dynamics and cognitive functions, and contribute to more reliable and accurate recognition performance in brain–computer interface applications [4].

Traditional methods for EEG signal reconstruction include interpolation, filtering, and time-series analysis. Interpolation techniques, such as linear or cubic spline interpolation [5], aim to estimate missing values by considering the neighbouring data points. While effective in some cases, these methods often oversimplify the complex nature of EEG signals and may introduce inaccuracies, especially in the presence of irregular patterns or abrupt changes. Filtering approaches [6] aim to eliminate noise and artefacts, but they may inadvertently alter the underlying signal or fail to address the non-linear characteristics of EEG data. Time-series analysis methods, such as autoregressive modelling [7], have also been utilised, but they may struggle to capture the intricate dynamics of brain activity.

Recent advancements in machine learning, particularly neural networks, have revolutionised EEG signal reconstruction. Deep learning models, such as convolutional neural networks (CNNs) and recurrent neural networks (RNNs), have demonstrated remarkable capabilities in capturing intricate temporal and spatial dependencies within EEG signals [8]. Unsupervised learning paradigms further contribute to EEG signal reconstruction when labelled data are scarce and demonstrate flexibility in uncovering diverse patterns, rather than relying on predefined classes. Among the unsupervised learning models, autoencoders (AEs) have demonstrated a strong capacity for inferring latent information and learning complex representations of EEG data [9,10], enabling them to effectively recover EEG signals with higher fidelity.

In our previous study, we proposed the weighted gate layer autoencoder (WGLAE) [11], which is an AE architecture with gate control regularisation for relationship learning. The WGLAE learns to approximate relationships among multiple variables simultaneously through a single model. Specifically, it introduces a gate layer to control the input stream being fed into the AE learner and a weight layer to adjust the update of the reconstruction error according to the importance of the variables. The experimental results demonstrated its effectiveness in reconstructing EEG signals and missing channels. However, our previous work on the WGLAE had a limitation: it only applied a sequential control scheme to turn on and off each input variable/channel sequentially, without considering the physical meaning of the locations of the EEG sensors. It remains an open research question whether the adoption of different gate control mechanisms impacts the learning outcome. Our hypothesis is that having a control strategy that embeds the physical locations of EEG sensors can further enhance signal reconstruction performance.

The integration of EEG electrode positions into the WGLAE model’s learning process is grounded in well-established neurophysiological principles. EEG signals are generated by synchronised neural activity, which propagates through the scalp via volume conduction, causing spatially proximate electrodes to capture overlapping activity from underlying brain regions. Functional brain networks also exhibit spatial organisation, where neighbouring regions often participate in related processes (e.g., occipital electrodes in visual tasks or central electrodes in sensorimotor rhythms). This spatial dependency is leveraged in neurophysiological methodologies, such as source localisation, spatial filtering, and functional connectivity studies. These principles justify our hypothesis: electrode proximity correlates with functional similarity, enabling models to exploit spatial relationships for reconstruction. In this study, we focus on evaluating the impact of the gate control strategies of the WGLAE on EEG signal reconstruction.

The main contributions of this paper are summarised as follows:

- We propose gate control mechanisms that take into account the underlying physical locations of EEG sensors for regularising the learning process of a deep learning model in learning the inter-channel dependencies of EEG signals.
- We investigate the gate control mechanisms, including four mechanisms, under different experimental settings. The analysis confirms the impact of gating regularisation on the learning outcomes. Specifically, with the gating sequence embedding the sensors’ physical locations, the WGLAE is able to reconstruct EEG signals with higher accuracy, fidelity, and efficiency.

## 2. Related Works

Autoencoders, which are a subfamily of neural networks aimed at learning compressed representations of input data via encoding–decoding layers, are considered very useful for different machine learning tasks, including feature extraction, dimensionality reduction, and learning generative models. The architecture of autoencoders can be thought of as a simple feedforward neural network, where the size of both the input layer and output layer is the same. An AE consists of an encoder Y=f(X) that generates a latent representation and a decoder Z=g(Y) that decodes this latent representation back to the original representation space. The objective is to minimise the difference between the final representation *Z* and the original input X using a loss function that captures this distance as an error loss(X,g(f(X)). The loss functions are the same as those used by the neural network, i.e., MSE, RMSE, MAE, etc. The size of the middle layer determines whether it is an under-complete autoencoder or an over-complete autoencoder. An under-complete autoencoder is used to overcome the potential of an AE learning to copy the input to the output g(f(X))=copy(x), where it does not learn any significant information. Regularised autoencoders constrain what the autoencoder can learn, where additional constraints are applied to guide the learning process.

Each regularised AE uses specific terms crafted to control the properties of the generated representation. Sparse AEs [12,13] add the sparsity term (h) to impose a sparsity penalty in the loss function loss(x,g(f(x)))+λΩ(h). The sparsity penalty $\Omega \left(h\right)=\sum_{i}\left|h_{i}\right|$ is computed using a Laplace prior. Contractive AEs [14] penalise the representation’s sensitivity to the input data using the Frobenius norm of the Jacobian matrix, which generates a localised space contraction with loss(x,g(f(x)))+λΩ(h), where $\Omega \left(h\right)=\lambda {∥\frac{\partial f(X)}{\partial X}∥}_{F}^{2}$. Variational AEs [15] use the Kullback–Leibler divergence principle Ω(h)=KL[Q((z∣x))||P(z)] with loss(x,g(f(x)))+λΩ(h) to learn to minimise the difference between the learnt representation distribution and the original sample space distribution. Denoising AEs [16] aim to minimise the distance between the noised input $\tilde{x}$ and its original form *x* using a loss function $loss(x,g\left(f\left(\tilde{x}\right)\right)$.

The GLAE [10] and WGLAE [11] use an input gate layer to guide the autoencoder to learn simultaneous multi-function approximation. This facilitates learning inter-relationships for every variable in the dataset, which can be a complex and time-consuming task for high-dimensional datasets. Both the GLAE and WGLAE have shown superior incomplete data recovery for EEG data compared to CAEs, DAEs, and VAEs when tested using different datasets. Both the GLAE and WGLAE share the same underlying architecture, which gates out the target variable to enforce learning its estimation using the remaining variables. This process is repeated systematically to enable learning for all variables. Therefore, the same sample can be seen multiple times, but with a different target variable each time.

The GLAE and WGLAE architectures enable a systematic teaching process that considers the full training dataset as a curriculum and structures the learning into lessons and sessions, as will be explained further in the next section. A single session is a single pass of a sample with a specific dependent variable gated. A lesson consists of sessions, and the lesson is completed when the number of sessions allows the network to see the same sample the required number of times to learn, approximating every single variable in this sample. However, the original architectures only apply a random sequence scheme without taking into account the physical locations of EEG electrodes. The order of sequential signals is critical due to the temporal structure, while the order of non-sequential signals can be significant in the spatial aspect. As EEG signals reflect both temporal and spatial aspects, we hypothesise that they impact learning performance. Many machine learning methods assume a certain level of order [17] without exploring the actual influence of the data order on performance. Therefore, we will focus on the gate control mechanisms in this study.

In addition to the AE family, recent advances in EEG reconstruction include diffusion models [18] for super-resolution, although their computational demands hinder real-time use, and GAN-based approaches [19,20], which achieve high fidelity but suffer from training instability and artefact sensitivity. Meanwhile, spatiotemporal correlation methods [21] reconstruct missing channels via fixed spatial heuristics, limiting their adaptability to dynamic neural interactions. In contrast, our WGLAE framework can uniquely integrate neurophysiological priors (e.g., electrode proximity and hemispheric symmetry) into lightweight, spatially informed gating mechanisms, thereby balancing efficiency and interpretability. By learning adaptive dependencies through task-specific gates, the WGLAE avoids the computational overhead of diffusion models, the instability of GANs, and the rigidity of correlation-based interpolation, positioning it as a pragmatic solution for real-time EEG applications requiring biologically plausible reconstruction.

## 3. Methodology

This section provides a brief description of the WGLAE model and proposes layout-inspired gate control mechanisms for it.

### 3.1. Weighted Gate Layer Autoencoder (WGLAE)

The gate layer autoencoder (GLAE) [10] was introduced to address the limited ability of conventional autoencoders to explicitly model inter-variable dependencies within multivariate data. Standard autoencoders, whether classical or regularised, treat all input dimensions equally and reconstruct the input vector holistically, thereby lacking an inductive bias towards learning functional relationships among variables. The GLAE augments the autoencoder architecture with an input gate layer controlled by a binary mask, allowing the selective suppression of input variables during training. The variables masked in each training iteration are treated as the dependent variables (DVs), while the unmasked variables serve as the independent variables (IDVs) from which the model must infer the DVs. This targeted masking strategy enables the autoencoder to approximate variable-level conditional mappings, promoting the discovery of inter-variable structure. The GLAE organises the training process into hierarchically structured iterations consisting of sessions and lessons. A session corresponds to a single training pass with a specific subset of input variables gated off. A lesson comprises multiple sessions and is completed once every variable in the input vector has been gated and reconstructed at least once. This mechanism ensures comprehensive exposure of the model to the full set of conditional estimation tasks embedded in the dataset. Unlike prior models referred to as “gated autoencoders”, which rely on multiplicative gating and were derived from extensions of Boltzmann machines, the GLAE introduces gating at the architectural level to structure learning. Empirical studies, particularly in EEG signal recovery, have demonstrated the GLAE’s efficacy in reconstructing missing data and capturing latent variable dependencies, with consistent improvements over classical autoencoder variants, including vanilla AEs, denoising AEs, and the variational GLAE [10].

Building on the foundational structure of the GLAE, the weighted gate layer autoencoder (WGLAE) [11] introduced a generalised framework that incorporates variable-specific weighting into both the gating and learning processes. While the GLAE applies uniform binary gating to mask variables, the WGLAE integrates a dynamic weighting mechanism that modulates the contribution of each variable to the reconstruction loss. This is achieved through the addition of two key modules: a weight controller and an error-weight generator. These modules assign distinct weights to DVs and IDVs during training, enabling the model to prioritise learning relationships that are considered more informative or relevant based on task-specific criteria. The WGLAE training pipeline is governed by three synchronised control sequences: (i) data control, which schedules training samples; (ii) gate control, which defines the binary masking of input variables; and (iii) weight control, which determines the loss contribution of each variable. This structure enables session-level modulation of both the input space and the learning signal. Furthermore, the WGLAE introduces a temporal decay mechanism for the weights of dependent variables (DVs), allowing the network to initially emphasise challenging estimation targets before gradually normalising their influence. Through this flexible and adaptive training scheme, the WGLAE achieves improved performance in variable reconstruction, particularly under conditions of structured or large-scale missingness. Comparative evaluations on synthetic and real EEG datasets have confirmed the superiority of the WGLAE over the GLAE and other baseline autoencoder architectures in both reconstruction fidelity and generalisation capacity [11]. Furthermore, the WGLAE was employed as a benchmark in a 2024 study on spatial super-resolution using spatiotemporal diffusion models [18], where its ability to reconstruct occluded channels was explicitly validated.

**Figure 1:**
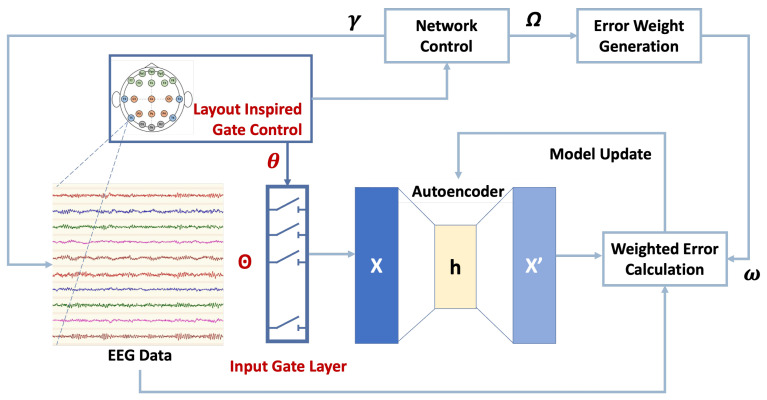
WGLAE model architecture, with the gate control module highlighted.

The WGLAE is an advanced autoencoder architecture designed to enhance dependency learning among input variables. Specifically, the GLAE introduces gate and network controllers, where the gate controller manages input data flow by switching variables ON or OFF using a binary gating sequence, treating switched-off variables as dependent variables (DVs) to be predicted from switched-on independent variables (IDVs). The network controller synchronises the gate and data control sequences, enabling the GLAE to learn variable dependencies beyond traditional data representations. The WGLAE extends the GLAE by incorporating a dynamic error-weighting mechanism, adding flexibility and accuracy in learning. It produces a weight sequence to regulate the error-weight generator, enabling more precise backpropagation by considering the importance of variables, making it suitable for adaptable applications. Figure 1 illustrates the complete architecture of the WGLAE, which includes the following four modules:

- Input Gate Control Module: This module generates a binary gate control sequence $\theta^{j}$, which is applied to the input layer via element-wise multiplication, forming the gated input:
  (1) $${\mathrm{xg}}_{i}^{j}=x_{i}⊙\theta^{j}\;.$$
  Each element of $\theta^{j}$ controls whether the corresponding input variable is activated or deactivated. The deactivated variables are set to zero and excluded from the forward path during training.
- Network Control Module: This module orchestrates the learning process by generating and synchronising three control sequences: the gate control $\theta^{j}$, the data control $\gamma^{j}$, and the weight control $\Omega^{j}$. These sequences determine which variables serve as inputs or targets (IDVs/DVs) and assign corresponding weights. The network dynamically updates these roles across training iterations to enhance the model’s ability to learn inter-variable dependencies.
- Error Weight Generation Module: The weight control sequence $\Omega^{j}$ includes the error weights $\omega^{j}$, which are applied to modulate the contribution of each variable in the loss function. Specifically, $\omega^{j}$ decays the influence of the DVs during training, adjusting their learning impact adaptively.
- Error Calculation Module: The reconstruction ${\hat{x}}_{i}^{g}$ is computed via feedforward propagation using the gated input. Using a sigmoid activation function and a single-layer encoding, the reconstructed variable can be computed using:
  (2) $${\hat{\mathrm{xg}}}_{i}^{j}=\sigma \left(\sum W_{ho}\cdot \sigma \left(\sum W_{ih}\cdot \left(x_{i}⊙\theta^{j}\right)\right)\right)\;.$$
  The weighted reconstruction error is then calculated using:
  (3) $${\mathrm{wLoss}}_{i}^{j}=\sqrt{\frac{\sum_{k=1}^{N_{v}}\omega_{k}^{j}\cdot {\left({\hat{x}}_{i,k}^{j}-x_{i,k}\right)}^{2}}{N_{v}}}\;.$$
  This error is used in backpropagation to update the network parameters.

Refer to the original paper [11] for detailed descriptions of the model and algorithm. In this study, we focus on the input gate control module and investigate different gate control mechanisms.

### 3.2. Gate Control Mechanisms

In the original WGLAE model [11], a random sequence gate control scheme was adopted in the input gate layer. That is, the DVs were selected randomly without considering any dependencies either within the DVs or between the DVs and IDVs. This random scheme demonstrates the resilience of the WGLAE model. However, it may not be able to fulfil the potential of the WGLAE model, thus making it less efficient in applications. Inspired by developments in neuroscience, three gate control mechanisms are proposed—order-based, partition-based, and hemispheric symmetry rotation gate control mechanisms—in addition to the random scheme adopted in the original WGLAE model [11]. While the random scheme applies a random permutation, the three proposed gate control mechanisms incorporate the order, partition, and hemispheric symmetry features of brain signals corresponding to EEG electrode placement.

From a machine learning perspective, integrating EEG electrode positions into the gating mechanism of our model is motivated by structured inductive bias [22], a fundamental concept in machine learning, where prior knowledge about data structure guides model design to improve generalisation. EEG signals exhibit spatial dependencies due to volume conduction (where signals from a neural source propagate to multiple nearby electrodes) and functional correlations between adjacent brain regions. By encoding electrode locations into the gating mechanism, we explicitly introduce a spatial prior that reduces the hypothesis space and enables efficient feature interaction. While neurophysiological studies support the spatial nature of EEG, our work demonstrates that explicitly encoding the spatial structure as an inductive bias enhances model robustness and efficiency in a purely data-driven setting.

#### 3.2.1. The Order-Based Gate Control Mechanism

Brain lateralisation, also known as hemispheric specialisation, refers to the functional dominance of one hemisphere of the brain [23]. It is involved in many advanced neural functions, including cognitive performance, memory, and handedness [23]. As pointed out in [24], brain asymmetries are significant to the functioning of the sensory, cognitive, and motor systems in humans and other animals. For instance, when conducting analytical or logical tasks, active beta waves can be observed in the left hemisphere. Therefore, it is fair to assume that EEG electrodes located at the left scalp region exhibit stronger interdependence than those at the right scalp region. For this reason, it is essential to learn the dependencies of EEG signals in the left brain hemisphere on those in the right hemisphere. Additionally, EEG electrodes generally have a stronger influence on nearby electrodes than on those farther away. Inspired by this idea, the order-based gate control mechanism first selects the DVs corresponding to the EEG electrode(s) located at the front-left scalp region, followed by those in the middle-left and back-left regions, and then applies the same sequence on the right scalp region.

**Figure 2:**
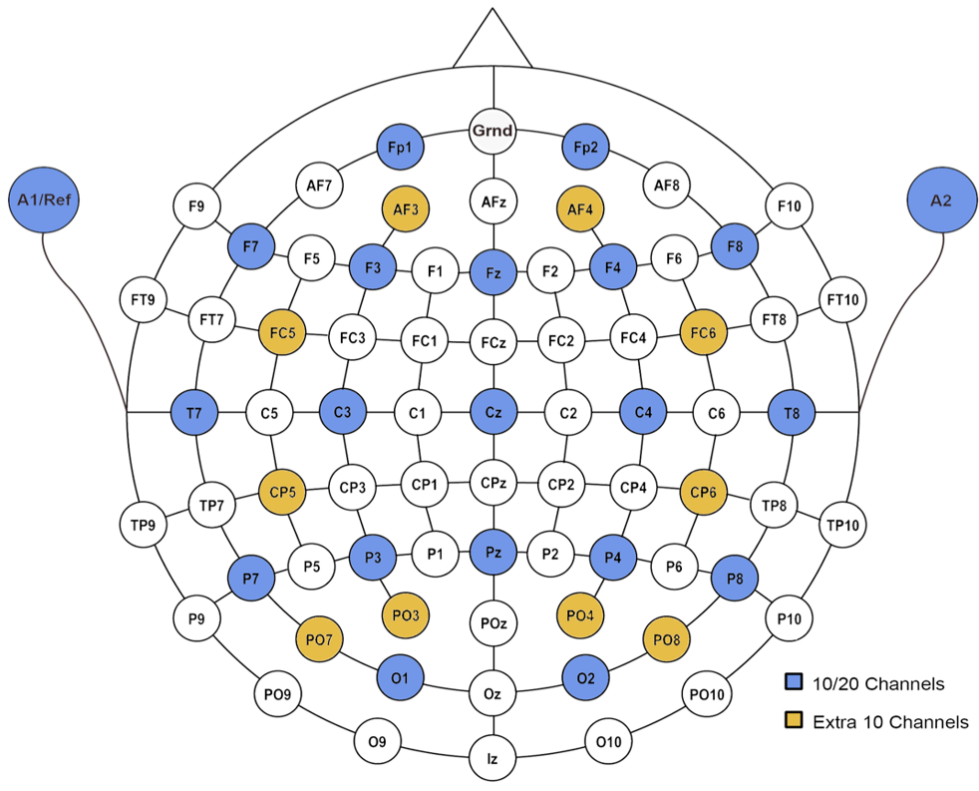
Head map of EEG scalp electrode placements [25].

For a clearer explanation, the order-based gate control mechanism is explained using the international EEG scalp electrode system. Figure 2 shows a head map of EEG electrode placements derived from the international 10-10 system and international 10-20 system (blue circles). In the international 10-10 system, there are 64 EEG electrodes in total, whereas in the international 10-20 system, there are 19 electrodes. Each electrode is labelled based on the lobe and specific area of the brain it records. The lobe location is indicated by letters and numbers. For letters, *Fp* stands for fronto-polar, *F* for frontal, *C* for central, *T* for temporal, *P* for parietal, and *O* for occipital. The numbers denote lateralisation. In particular, electrodes located on the right brain hemisphere are indicated by even numbers, while those on the left brain hemisphere are indicated by odd numbers.

**Figure 3:**
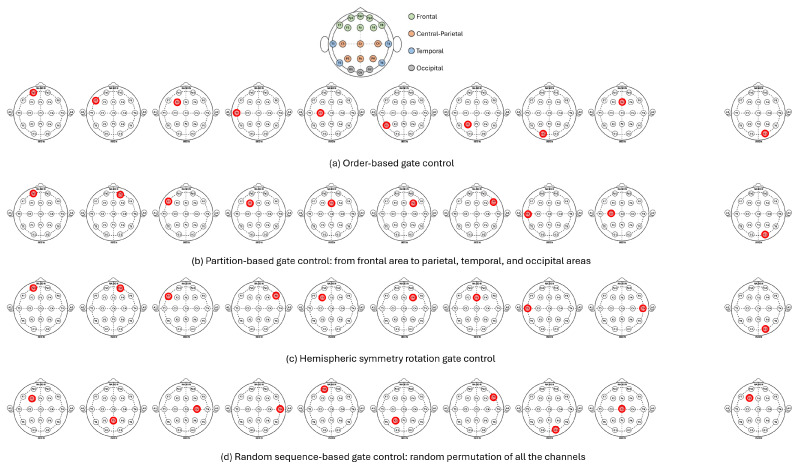
Illustration of gate control sequences generated by the four gate control mechanisms on the 19-channel dataset.

As shown in the head map, the order-based gate control mechanism selects DVs from the top-left section to the bottom-left section, followed by the top-right and bottom-right sections. The mechanism is exemplified in Figure 3a. A full list of the order-based sequence, based on the international 10-20 system, is presented in Table 1.

#### 3.2.2. The Partition-Based Gate Control Mechanism

A human’s cerebral cortex is divided into different lobes, as illustrated in the legend of Figure 3. Each cortex lobe is responsible for processing certain information and is associated with specific functions [26]. Importantly, the pattern of brain waves can be closely related to neural functions. For instance, gamma-band oscillations (30–80 Hz) play a crucial role in attention and consciousness [27]. Assuming that brain signals located in the same lobe are more dependent on each other, it is expected to be more efficient to learn the relationships among signals generated by different cortex lobes. Inspired by this assumption, the proposed partition-based gate control mechanism sets the sequence of DVs based on the cortex lobes.

In the partition-based gate control mechanism, the channels at the fronto-polar lobe are set as the DVs, and their dependency on the channels associated with the other cortical lobes is learned first, followed by the channels located at the frontal and central lobes. The channels located at the parietal and occipital lobes are learned last. A complete list of the sequence based on the 19-channel EEG electrode system is presented in Table 1. With the partition-based gate control mechanism, channel dependency within and across lobes is learned when very few channels are missing, and the dependency of channels in one lobe on those in other lobes is learned when more channels are missing.

#### 3.2.3. The Hemispheric Symmetry Rotation Gate Control Mechanism

Despite its functional asymmetry, the human brain is a structurally mirrored organ [28] and inherently displays bilateral symmetry [23]. In certain circumstances, such as a restful state with closed eyes, brain waves can show symmetrical patterns of activity across the hemispheres [29]. This symmetrical brain feature promotes the efficiency of data representation, which further improves the capability for information processing and responding [30].

Considering the anatomical symmetry of the left and right hemispheres of the brain [23], it can be assumed that the brain waves captured by EEG electrodes located in symmetrical regions have a stronger correlation. Accordingly, the hemispheric symmetry rotation gate control mechanism explores the feasibility and benefit of learning channels based on symmetry. As depicted in Figure 3c, the gate control sequence starts from the EEG electrode located at the fronto-polar region on the left side, followed by the symmetrical EEG electrode on the right side.

Similar to the partition-based gate control mechanism, the gate control sequence in the hemispheric symmetry rotation mechanism is based on the cerebral cortex areas. However, within each cortical area, the hemispheric symmetry rotation mechanism prioritises symmetry over proximity. The full channel sequence of the hemispheric symmetry rotation mechanism, based on the international 10-20 system, is exemplified in Table 1. It can be seen from the EEG electrode labels that even numbers and odd numbers appear alternately. This is because a left EEG electrode is always followed by the electrode placed either on the right side or in the central area in this mechanism.

#### 3.2.4. Summary

The gating mechanism in our WGLAE framework functions as a structured form of regularisation, explicitly encoding domain-specific priors to guide the model towards learning neurophysiologically meaningful dependencies. By constraining interactions based on spatial proximity (e.g., partition-based gates), functional symmetry (e.g., hemispheric gates), or task relevance, the gate introduces inductive biases that reflect neurophysiological principles such as volume conduction and modular brain networks. This structured sparsity suppresses noisy or implausible correlations, reduces overfitting, and prioritises neurophysiologically meaningful relationships. Empirically, gates with such priors outperform unstructured variants [31], validating their role in enhancing robustness and generalisability. Thus, the gate operates not merely as a control mechanism but as a neurophysiologically grounded regulariser, bridging data-driven learning with domain knowledge to ensure biologically plausible signal reconstruction.

## 4. Experimental Design

### 4.1. Database and Pre-Processing

The proposed method was evaluated using the publicly available EEG Motor Movement/Imagery Database (MMIDB) [32]. The MMIDB contains EEG recordings from 109 healthy subjects under various signal elicitation protocols [33], including resting states with eyes open and closed, motor-movement tasks involving opening and closing fists or feet, and motor imagery tasks requiring subjects to imagine these movements. This database is widely used in EEG classification studies due to its diversity in subject count and recording conditions [34,35]. The EEG signals were recorded using the BCI2000 system [36], which features 64 scalp-mounted sensors following the international 10-10 system. The system operated at a 160 Hz sampling rate, using the earlobes as signal references.

For EEG signal pre-processing, we implemented an automated pipeline consisting of filtering, artefact removal, and segmentation. First, the raw EEG data were band-pass filtered between 0.5 Hz and 42 Hz to preserve the canonical EEG frequency range. Next, independent component analysis (ICA) and the Multiple Artefact Rejection Algorithm (MARA) [37] were applied to eliminate artefacts caused by eye movements, muscle activity, and sensor contact issues. Finally, a non-overlapping sliding window was used to segment the continuous signals into two-second segments.

While the MMIDB provides a controlled benchmark for evaluating gating mechanisms, future studies will test the WGLAE’s generalisability on clinical EEG datasets with diverse pathologies and noise profiles. The framework’s integration of neurophysiological priors positions it as a promising candidate for cross-dataset adaptation, particularly when combined with proper domain adaptation strategies.

### 4.2. Experiments

In this paper, the WGLAE with the different gating control mechanisms was used to learn the relationships between EEG channels. In order to investigate how the physical locations of EEG channel(s) affect the learning capability of the WGLAE, the EEG channels were blocked in different sequences and in different quantities when training the WGLAE. Then, the trained WGLAE model was used to reconstruct the EEG data with incomplete channels. The proposed approach was evaluated through the following: an evaluation of the impact of gate control mechanisms on reconstruction performance, a scalability analysis, and an evaluation of reconstructed EEG in classification tasks.

In the reconstruction performance experiment and the scalability analysis, the parameter settings were the same for all datasets to ensure fair comparisons. The WGLAE model consisted of a single hidden layer, with the number of neurons set to half the input size to build an under-complete structure of the AE. Based on the preliminary work in [11], the WGLAE model was configured as follows: the activation function was sigmoid, the optimiser was ADAM [38], the learning rate was 0.00001, the total number of epochs was 10,000, and the batch size was 1024.

The data setup of the WGLAE model followed the widely used 80% to 20% ratio. That is, for each dataset, the training data accounted for 80% of the total data, while the testing data accounted for 20%. In addition, to avoid overfitting, 20% of the training data were randomly selected to serve as validation data. The same training, validation, and testing datasets were employed across all four gating strategies.

The training and learning process conformed to the curriculum, session, and lesson framework proposed in [11]. In a curriculum, a specific gate control mechanism is applied, and the number of missing channels is fixed. Within one curriculum, each session is assigned one data sample, under which different lessons are in charge of different locations of the missing channels (DVs). Therefore, the number of sessions in one curriculum equals the number of samples, and the WGLAE learns multiple sessions simultaneously. The number of lessons $N_{lesson}$ in one session is determined by the number of channels $N_{DV+IDV}$ and the number of missing channels $N_{DV}$:

(4) $$N_{lesson}=\left\lfloor N_{DV+IDV}/N_{DV}\right\rfloor$$

where ⌊⌋ is the floor function. Reconstruction performance was evaluated based on the differences between the reconstructed data and the original complete data, and the average reconstruction errors of 10 runs were employed.

#### 4.2.1. Impact of Gate Control Mechanisms on Reconstruction Performance

The first set of experiments evaluated the reconstruction errors of WGLAE models trained with different gate control mechanisms. The reconstruction errors from the three proposed mechanisms, i.e., partition-based, hemispheric symmetry rotation, and order-based, were compared with those from the random sequence gate control mechanism. This experiment assessed how different gate control strategies influence the accuracy and quality of EEG signal reconstruction, providing insights into their effectiveness in modelling inter-channel dependencies.

To evaluate the reconstruction performance of the three mechanisms under different scenarios, the experiments were conducted based on two EEG electrode configurations: the international 10-20 system, where 19 channels of signals were considered, and the extended system, where 29 channels were examined. The EEG electrode placements for the two configurations are presented in Figure 2, where the blue circles indicate the configuration based on the international 10-20 system, and the yellow circles denote the additional 10 channels of the extended system. The comparison results are expected to enhance our understanding of the correlations among EEG channels.

#### 4.2.2. Scalability Analysis

The scalability analysis aimed to examine the influence of the number of blocked channels (DVs) on the reconstruction error. When more channels are blocked, the WGLAE receives less ‘supervision’ during training, and thus learns relatively less about the EEG data. However, a resilient model should still maintain a certain level of reconstruction accuracy. The scalability analysis evaluated the model across varying numbers and configurations of missing channels, demonstrating its adaptability to different EEG electrode configurations and real-world applications.

#### 4.2.3. Evaluation of Reconstructed EEG in Classification Tasks

The reconstruction error reflects the discrepancy between the original signal and its reconstructed version in the time domain, providing a measure of the accuracy and fidelity of the reconstruction process. While the reconstruction error is a valuable metric for evaluating signal reconstruction performance, it offers limited insight into data patterns and biological relevance [39]. For example, the reconstruction error does not inherently capture whether the reconstructed signal retains meaningful information about neural processes or cognitive states. Hence, in this experiment, we evaluated the usability of EEG data reconstructed under different gating mechanisms using a classification task: biometric identification [34,40]. The Motor Movement/Imagery Database was used for this evaluation.

Classification performance can reflect the extent to which the reconstructed data capture the underlying patterns identified by the learning model in the original data. In this task, we classified EEG signals into 109 classes corresponding to the identities of the 109 subjects. A convolutional neural network (CNN) model was trained using 80% of the original data. For the remaining 20% of testing data, we randomly discarded one, five, and ten channels and replaced them with reconstructed data using the proposed method to simulate scenarios where certain channel data are lost in real applications. Performance was evaluated using the Correct Recognition Rate (CRR), which indicates the classification accuracy. The details of the classification model configurations are summarised in Table 2. The training process used the ADAM optimiser with a learning rate of 0.0005, a batch size of 32, 200 training epochs with early stopping, batch normalisation, and a dropout rate of 0.1 after each pooling layer.

## 5. Experimental Results Analysis

### 5.1. Reconstruction Accuracy

The reconstruction accuracy evaluates the credibility of the proposed WGLAE model by calculating the mean squared difference between the original and reconstructed signals. The performance of the three gate control mechanisms, as well as the random sequence gate control mechanism, was examined based on the international EEG placement system with 19 channels. The MMIDB with three elicitation protocols, including resting states, motor imagery, and motor movement, was employed.

The reconstruction errors across the entire sample are presented in Table 3, and the best-performing gate control mechanism in each case is highlighted. For all the examined gate control mechanisms and datasets, the reconstruction accuracy decreased as more channels were blocked. This aligns with real-world experiences; that is, as missing information increased, it became more challenging to reconstruct EEG signals. Another common phenomenon observed across all three datasets was that the three proposed gating mechanisms performed better than the random sequence gating mechanism in most cases. This indicates the necessity of considering EEG electrode placement when designing algorithms for signal reconstruction.

On the other hand, the results demonstrated the various influences that the three gate control mechanisms had on the reconstruction performance of the WGLAE. On the resting-state and motor-imagery datasets, the hemispheric symmetry rotation gate control mechanism outperformed the other three mechanisms in 9 out of 10 different quantities of missing channels. This observation can be explained by the brain’s alpha waves, which are active in states of relaxed and passive attention [29]. Particularly in an eyes-closed state, alpha waves were typically balanced between the left and right hemispheres, suggesting similarities and dependencies between the EEG signals captured by the left electrodes and those captured by the right electrodes. As the hemispheric symmetry mechanism aims to learn EEG signals obtained from symmetrical areas, it should facilitate better learning capabilities for the WGLAE. A different trend was observed in the motor-movement dataset, where the partition-based gate control mechanism exhibited the best performance in 8 out of 10 cases. This could be due to the active beta and gamma waves in the brain, which are closely related to movement execution and coordination. As beta waves and gamma waves show dominance in various cerebral areas when undertaking different tasks, learning the dependencies between different cerebral cortex lobes can be significant for building an effective EEG signal reconstruction model. The advantages observed in the hemispheric symmetry rotation and partition-based gate control mechanisms validate the benefits of employing gate control mechanisms. The disadvantage of the hemispheric symmetry rotation gate control mechanism can be justified by the lateralisation of motor functions such as handedness [23].

The results suggest that the application context should be taken into account when selecting gate control mechanisms. Specifically, in scenarios involving resting states, the hemispheric symmetry rotation gate control mechanism should be prioritised, while the partition-based gate control mechanism is more suitable when motor movement is involved. The observed differences in the efficacy of the gating mechanisms across tasks can be attributed to distinct functional brain connectivity patterns associated with resting states versus motor movements. Resting-state EEG was characterised by synchronised activity within default mode networks, which spanned the medial prefrontal and posterior regions. These networks exhibited strong long-range and inter-hemispheric coherence, reflecting idling cortical activity. The hemispheric symmetry rotation gate control mechanism, which explicitly models interactions between homologous brain regions (e.g., left vs. right motor cortex), aligns with this bilateral synchronisation. By emphasising cross-hemispheric dependencies, this gate effectively captured the large-scale functional connectivity inherent to resting states. On the other hand, motor tasks (e.g., hand movement) engaged localised, task-specific networks primarily within the motor cortex. These regions exhibited short-range and intra-hemispheric coherence. The partition-based gate control mechanism, which groups electrodes into localised clusters (e.g., frontal, central, or occipital), prioritised short-range spatial relationships. This design mirrored the organisation of the motor cortex, where adjacent electrodes captured activity from functionally related representations, enhancing the reconstruction of task-specific signals. The proposed gating strategies aligned with the neural mechanisms. Specifically, the hemispheric symmetry rotation gate control mechanism acted as a coarse-grained prior for global connectivity, while the partition-based gate control mechanism imposed a fine-grained prior for local connectivity. Their task-dependent efficacy suggests that the model successfully leveraged the spatial structure of functional networks in a data-driven setting.

The standard deviations in some cases were relatively high, for example, when reconstructing 10/19 channels for motor-movement data. An analysis of the extreme cases revealed the context-dependent efficacy of the gate mechanisms. For example, the partition-based gate mechanism exhibited larger deviations when critical task-relevant channels (e.g., C3 for right-hand motor imagery) were blocked, underscoring the need for adaptive strategies in future work.

**Figure 4:**
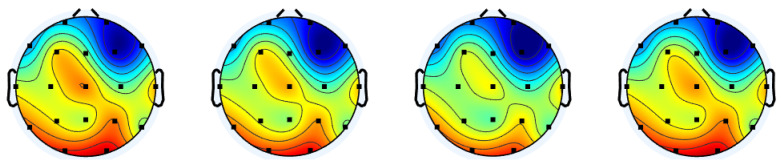
Reconstruction error distributions over the scalp (resting state). The corresponding gate control mechanisms, from left to right, are hemispheric symmetry rotation, partition-based, order-based, and random.

To understand how the different gate control mechanisms influenced the reconstruction accuracy in cerebral cortex regions, the reconstruction error distributions over the scalp are presented in Figure 4. It can be observed that the central and posterior areas show higher errors compared to the frontal area, and this trend is consistent across the different gate control mechanisms. This can be explained by the brain functions associated with the regions of the cerebral cortex. Specifically, the frontal lobe is responsible for executive functions and motor control [41], which are active from stimuli incorporated into the MMIDB. On the other hand, the occipital lobe is mainly in charge of visual processing [41], which might not have been intensively activated by the three elicitation protocols.

To assess spectral fidelity, we conducted post hoc frequency-domain analyses to validate spectral preservation. These analyses focused on quantifying the fidelity of oscillatory dynamics across the five canonical EEG frequency bands: delta (1–4 Hz), theta (4–8 Hz), alpha (8–13 Hz), beta (13–30 Hz), and gamma (30–50 Hz). Specifically, we computed the power spectral density (PSD) for both the original and reconstructed signals using Welch’s method and estimated band-specific power. Then, Pearson’s correlation coefficients were calculated between the PSD of the original and reconstructed signals across all channels and subjects to assess the global spectral alignment. The normalised RMSE (nRMSE) was computed for each frequency band to quantify the deviations in the reconstructed band power. Paired *t*-tests were performed to evaluate the significance of the band-power differences between the gating strategies. The results of the spectral fidelity analysis are summarised in Table 4.

For the resting-state data, the WGLAE framework demonstrated better reconstruction performance using the hemispheric symmetry rotation gating scheme, achieving higher spectral correlation and lower spectral nRMSE compared to the random sequence gating scheme, with statistically significant differences (*p* < 0.05). Similarly, the partition-based gating scheme achieved better spectral fidelity than the random sequence gating scheme for motor-movement data reconstruction. Although marginal in absolute value, these improvements reflect meaningful noise suppression, particularly in middle- and high-frequency bands (alpha, beta, and gamma). The results show strong preservation of oscillatory dynamics and demonstrate the importance of neurophysiologically informed gating in capturing task-relevant spatial dependencies while mitigating noise. As many EEG features are in the frequency domain, spectral fidelity is important in downstream tasks such as sleep-stage classification, cognitive load assessment, and other BCI control applications. While our model applies time-domain metrics in the learning process, minimising amplitude errors inherently enforces implicit spectral fidelity. This is supported by studies showing strong correlations between time-domain reconstruction accuracy and spectral preservation in autoencoder-based EEG models [42].

While neurophysiological studies validate the spatial basis of EEG signals (e.g., interpolation of missing channels using splines), our work computationally confirms this by demonstrating improved reconstruction when spatial gates encode electrode locations. Our experimental results align with prior neurophysiological findings, showing that models incorporating spatial information outperform random approaches. Thus, our hypothesis is supported by both established literature and empirical validation in this study.

### 5.2. Scalability Analysis

The capabilities of the proposed gate control mechanisms were further tested on the MMIDB with 29 channels. The placement of the 29 channels is illustrated in Figure 2. In this section, the reconstruction accuracy obtained by different gate control mechanisms is discussed, followed by a comparison with the accuracy obtained on the dataset with 19 channels.

As presented in Table 5, the hemispheric symmetry rotation, partition-based, and order-based gate control mechanisms outperformed the random sequence gate control mechanism on 26, 25, and 26 out of 30 instances, respectively. Similar to the observations on the 19-channel dataset, the hemispheric symmetry rotation gate control mechanism performed the best on the resting-state dataset, while the partition-based gate control mechanism showed dominant advantages on the motor-movement dataset. This observation is similar to that on the 19-channel dataset, thus validating the benefits of aligning EEG sensor locations with the features of active brainwaves. The difference between the performance on the 19-channel and 29-channel datasets lies in the motor-imagery protocol. In this elicitation protocol, the partition-based gate control mechanism exhibited the best performance on the 29-channel dataset, while the hemispheric symmetry rotation gate control mechanism exhibited the best performance on the 19-channel dataset. Therefore, the features of datasets, especially the data size, should also be considered when deciding on the most fitting gate control mechanism.

**Figure 5:**
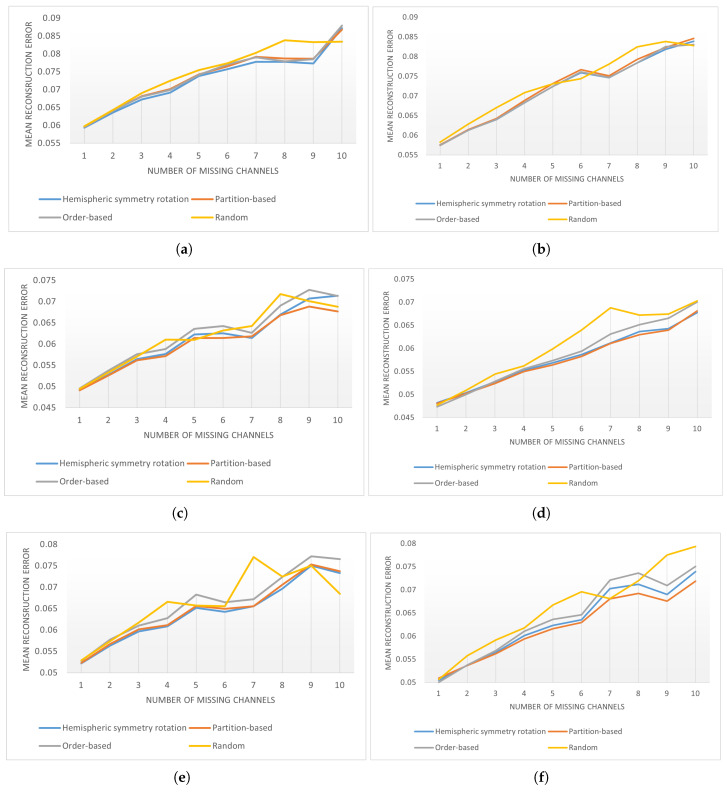
The mean reconstruction errors (RMSE) across all samples when reconstructing different numbers of missing channels. The left and right columns show the results from the 19- and 29-channel datasets, respectively. (**a**) 19 channels—Resting State; (**b**) 29 channels—Resting State; (**c**) 19 channels—Motor Movement; (**d**) 29 channels—Motor Movement; (**e**) 19 channels—Motor Imagery; (**f**) 29 channels—Motor Imagery.

For the scalability analysis, we evaluated the performance of the WGLAE model with different gate control mechanisms in reconstructing different numbers of channels from 1 to 10. The mean reconstruction errors on the 19-channel and 29-channel datasets are presented in Figure 5. With an increasing number of missing channels (DVs), the reconstruction errors from all four gate control mechanisms showed an upward trend. This upward trend is consistent with the observations on WGLAE models, where more input information generally yields a better model. At the same time, it is worth noting that the reconstruction accuracy was consistently higher than 91%, even when more than half of the EEG channels were blocked during WGLAE training. This shows the resilience and reliability of the WGLAE model in recovering real-world EEG signals. The advantages of the proposed gate control mechanisms over the random sequence gate control mechanism tended to decline with an increasing number of missing channels. This is because when many channels are blocked simultaneously, it is futile to consider the functions of the cortex lobes, as the channels in the lobe are completely missing. This is also the reason why this performance deterioration was less pronounced in the 29-channel instances.

To examine the statistical difference between the performance of the random sequence gate control mechanism and that of the hemispheric symmetry rotation, partition-based, and order-based gate control mechanisms, a Wilcoxon signed-rank test was conducted [43]. A holistic comparison was made based on the reconstruction errors obtained in 10 scenarios (with 1 to 10 missing channels) of 10 runs. The significance values in Table 6 show that both the hemispheric symmetry rotation and partition-based gate control mechanisms were significantly different from the random sequence gate control mechanism. However, the order-based gate control mechanism was not significantly different from the random sequence gate control mechanism in the 19-channel configuration, which can be explained by the limited information available compared to that in the 29-channel configuration. On the other hand, it aligns with the aforementioned observations and indicates that the hemispheric symmetry rotation and partition-based gate control mechanisms were more effective in capturing signal patterns.

With our hardware setup, a computer with an i7-8550U CPU and 8 GB of RAM, the computational time for reconstructing the 19-channel dataset was between 0.08 and 0.16 s and between 0.15 and 0.2 s for the 29-channel dataset during the inference stage. This inference speed aligns well with the requirements for efficient and low-latency deployment. The proposed model adopts a structured gating mechanism on a lightweight autoencoder backbone, which ensures fast forward passes, with inference time comparable to that of standard simple neural networks or linear models.

The experiments adopted 19- and 29-channel configurations, which are common in clinical and BCI applications. The WGLAE’s structured gating principles are theoretically scalable to high-density systems. In future work, we will evaluate the model on high-density setups and refine the gating mechanisms for high-channel configurations, including hierarchical partitioning and dynamic sparsity adaptation.

### 5.3. Classification Performance

The utility of the reconstructed EEG signals was examined by applying them to classification problems, validating the model’s practical effectiveness in downstream tasks. Table 7 summarises the classification results, which complement the reconstruction error results and provide a more comprehensive understanding of the quality and fidelity of the reconstructed data. An increased number of missing channels led to a decline in classification accuracy. This was expected, as missing channels reduce the available information and impair the model’s ability to extract meaningful patterns. The decline appears more gradual when fewer channels are missing (1 or 5) but becomes more pronounced as more data are lost. The results show the effect of the different gate control mechanisms on classification accuracy. Across all conditions, the partition-based and hemispheric symmetry rotation gate control mechanisms consistently performed better than the order-based and random sequence gate control mechanisms, which suggests that structured control over missing data can be beneficial. The performance gap between the different mechanisms increased as more channels were missing, which demonstrates the importance of a structured approach to handling missing data. The results suggest that the proposed gate control mechanisms can help mitigate the effects of missing EEG channels, making classification systems more robust.

While subject identification validates the retention of discriminative features, future work will test the WGLAE’s clinical efficacy by integrating reconstructed signals into applications such as seizure detection by leveraging open datasets such as CHB-MIT.

## 6. Conclusions and Future Work

To facilitate reliable EEG signal reconstruction, this study proposed various gate control mechanisms based on the effective WGLAE model [11]. By considering the physical locations of EEG sensors and the corresponding brain functions, the three proposed gate control mechanisms exploit different features of brain activities. In particular, the order-based gate control mechanism is inspired by the brain lateralisation phenomenon, the partition-based gate control mechanism considers various functions related to different cerebral lobes, and the hemispheric symmetry rotation gate control mechanism emphasises the largely inherent symmetry of the brain structure and brain wave patterns in certain circumstances. The incorporation of such preliminary knowledge about brain waves is expected to enhance the applicability and effectiveness of the WGLAE, hence contributing to the understanding of cognitive processes.

The three proposed gate control mechanisms were examined on the commonly used MMIDB with three signal elicitation protocols, i.e., resting states, motor movement, and motor imagery. The experimental results on the dataset with 19 EEG channels showed that the three proposed gate control mechanisms dominated the random sequence gate control in most instances, although the best mechanism varied for different protocols. The scalability analysis, based on the extended dataset with 29 EEG channels, further validated the effectiveness of gating regularisation in deep learning models. Across the 19-channel and 29-channel datasets, the hemispheric symmetry rotation gate control mechanism performed best in the resting-state dataset, while the partition-based gate control mechanism showed advantages in the motor-movement dataset. This can be explained by the different brain activity patterns for different stimuli. In addition, the reconstructed EEG signals were applied to classification problems. The consistent superiority shown by the partition-based and hemispheric symmetry rotation gate control mechanisms in classification problems further validates the effectiveness of the proposed gate control mechanisms.

In this study, a classical AE backbone was chosen to isolate the efficacy of the gating mechanisms. However, future work will explore advanced architectures (e.g., VAEs) and hybrid models that integrate gates with adversarial or variational components for enhanced performance and artefact suppression. Moreover, future research could explore adaptive mechanisms that dynamically update the gating strategies proposed in this study, based on signal characteristics or task demands. Such approaches, potentially leveraging attention or meta-learning frameworks, may further optimise reconstruction performance across diverse EEG paradigms. In addition, future iterations could integrate domain-specific channel weights into the gating mechanism to align the model with neurophysiological principles and enhance task-specific reconstruction fidelity.

In terms of evaluation, future work will evaluate patient-independent generalisation using leave-one-subject-out validation to assess robustness against anatomical and noise variability across individuals. The WGLAE’s structured gates, which encode neurophysiological priors (e.g., spatial proximity and symmetry), are theoretically positioned to enhance cross-subject generalisation by prioritising conserved neurophysiological principles rather than subject-specific artefacts. Empirical validation of this hypothesis remains a critical next step. Meanwhile, the model’s robustness against dynamic artefacts (e.g., muscle noise or electrode drift) in practical settings is worth further examination. It would also be interesting to extend the work to other physiological data, such as ECG and EMG. While this study focuses on EEG, the gating framework’s reliance on structured priors suggests applicability to these signals by adapting spatial and temporal constraints to their unique properties [44,45].

Our model’s ability to learn spatiotemporal dependencies positions it as a promising alternative to purely spatial (e.g., spline) or temporal (e.g., AR) interpolation, particularly for scenarios requiring joint reconstruction of multi-channel, time-continuous signals. While this study has focused on evaluating gate control mechanisms within the WGLAE framework, future work will include direct comparisons with classical EEG reconstruction methods (e.g., spline interpolation) to further validate the practical advantages of our approach in both spatial and temporal recovery tasks. The proposed model also offers promising potential for applications such as EEG channel density enhancement [46] and data augmentation [47], both of which have proven critical for improving the performance of machine learning models, particularly deep neural networks, in EEG classification tasks.

## Figures and Tables

**Table 1:** Electrode sequences under the three proposed gate control mechanisms.

Order-based	FP1	F7	F3	T7	C3	P7	P3	O1	Fz	Cz
	Pz	Fp2	F4	F8	C4	T8	P4	P8	O2	
Partition-based	Fp1	Fp2	F7	F3	Fz	F4	F8	T7	C3	Cz
	C4	T8	P7	P3	Pz	P4	P8	O1	O2	
Hemispheric symmetry rotation	Fp1	Fp2	F7	F8	F3	F4	Fz	T7	T8	C3
	C4	Cz	P7	P8	P3	P4	Pz	O1	O2	

**Table 2:** Classification model configurations.

Person Identification Task:						
Layers	Kernel Size	#Kernels/Neurons Ch.	MaxPool	Stride	Padding	BN/AF/DP
Conv_1_	3×3	64	[2, 2]	[1, 1]	[0, 0]	Yes/ReLU/Yes
Conv_2_	5×5	64	[2, 2]	[1, 1]	[0, 0]	Yes/ReLU/Yes
Conv_3_	5×5	64	[2, 2]	[1, 1]	[0, 0]	Yes/ReLU/Yes
FL	nul	128	nul	nul	nul	No/ReLU/No
FL	nul	109	nul	nul	nul	No/Softmax/No

BN: Batch normalisation; AF: Activation function; DP: Dropout; FL: Fully connected layer.

**Table 3:** The average and standard deviation of the reconstruction errors (RMSE) of entire samples when reconstructing 1 to 10 channels out of 19 channels.

**Resting State:**				
Missing channels	Hemispheric symmetry rotation	Partition-based	Order-based	Random
1	**0.059 ± 0.003**	0.060 ± 0.003	0.060 ± 0.003	0.060 ± 0.003
2	**0.064 ± 0.003**	0.064 ± 0.004	0.064 ± 0.004	0.064 ± 0.004
3	**0.067 ± 0.004**	0.068 ± 0.004	0.068 ± 0.005	0.069 ± 0.005
4	**0.069 ± 0.004**	0.070 ± 0.005	0.070 ± 0.005	0.072 ± 0.007
5	**0.074 ± 0.007**	0.074 ± 0.005	0.074 ± 0.007	0.075 ± 0.006
6	**0.076 ± 0.009**	0.077 ± 0.008	0.077 ± 0.009	0.077 ± 0.007
7	**0.078 ± 0.009**	0.079 ± 0.009	0.079 ± 0.010	0.080 ± 0.013
8	**0.078 ± 0.008**	0.079 ± 0.010	0.078 ± 0.007	0.084 ± 0.012
9	**0.077 ± 0.004**	0.079 ± 0.005	0.079 ± 0.005	0.083 ± 0.010
10	0.087 ± 0.018	0.087 ± 0.016	0.088 ± 0.018	**0.083 ± 0.007**
**Motor Movement:**				
Missing channels	Hemispheric symmetry rotation	Partition-based	Order-based	Random
1	0.049 ± 0.002	**0.049 ± 0.002**	0.050 ± 0.003	0.049 ± 0.002
2	0.053 ± 0.003	**0.053 ± 0.003**	0.054 ± 0.004	0.053 ± 0.003
3	0.056 ± 0.005	**0.056 ± 0.005**	0.058 ± 0.006	0.057 ± 0.004
4	0.058 ± 0.005	**0.057 ± 0.005**	0.059 ± 0.005	0.061 ± 0.005
5	0.062 ± 0.006	0.061 ± 0.006	0.064 ± 0.008	**0.061 ± 0.006**
6	0.063 ± 0.007	**0.061 ± 0.006**	0.064 ± 0.009	0.063 ± 0.005
7	**0.061 ± 0.004**	0.062 ± 0.005	0.063 ± 0.004	0.064 ± 0.005
8	0.067 ± 0.006	**0.067 ± 0.007**	0.069 ± 0.008	0.072 ± 0.012
9	0.071 ± 0.011	**0.069 ± 0.008**	0.073 ± 0.012	0.070 ± 0.010
10	0.071 ± 0.017	**0.068 ± 0.011**	0.071 ± 0.015	0.069 ± 0.008
**Motor Imagery:**				
Missing channels	Hemispheric symmetry rotation	Partition-based	Order-based	Random
1	**0.052 ± 0.002**	0.052 ± 0.002	0.053 ± 0.002	0.053 ± 0.002
2	**0.056 ± 0.004**	0.057 ± 0.004	0.058 ± 0.004	0.057 ± 0.003
3	**0.060 ± 0.005**	0.060 ± 0.005	0.061 ± 0.006	0.062 ± 0.004
4	**0.061 ± 0.006**	0.061 ± 0.006	0.063 ± 0.007	0.067 ± 0.010
5	**0.065 ± 0.005**	0.066 ± 0.006	0.068 ± 0.008	0.066 ± 0.007
6	**0.064 ± 0.003**	0.065 ± 0.003	0.066 ± 0.005	0.066 ± 0.005
7	**0.066 ± 0.004**	0.066 ± 0.004	0.067 ± 0.004	0.077 ± 0.013
8	**0.070 ± 0.008**	0.071 ± 0.009	0.072 ± 0.010	0.072 ± 0.009
9	**0.075 ± 0.012**	0.075 ± 0.012	0.077 ± 0.013	0.075 ± 0.015
10	0.073 ± 0.022	0.074 ± 0.022	0.077 ± 0.028	**0.068 ± 0.007**

The best performance among the four gating mechanisms is highlighted in bold.

**Table 4:** Spectral fidelity analysis results (5 out of 19 missing channels).

**Reconstructed Resting State Data**		
Frequency band	Spectral correlation (Hemispheric vs. Random)	nRMSE (Hemispheric vs. Random)
Delta (1–4 Hz)	0.83 ± 0.04 (H) vs. 0.79 ± 0.05 (R) *	0.11 ± 0.02 (H) vs. 0.13 ± 0.03 (R) *
Theta (4–8 Hz)	0.86 ± 0.03 (H) vs. 0.81 ± 0.04 (R) *	0.10 ± 0.02 (H) vs. 0.12 ± 0.03 (R) *
Alpha (8–13 Hz)	0.90 ± 0.04 (H) vs. 0.85 ± 0.05 (R) *	0.06 ± 0.01 (H) vs. 0.09 ± 0.02 (R) *
Beta (13–30 Hz)	0.82 ± 0.05 (H) vs. 0.78 ± 0.06 (R) *	0.12 ± 0.02 (H) vs. 0.14 ± 0.03 (R) *
Gamma (30–50 Hz)	0.75 ± 0.06 (H) vs. 0.72 ± 0.07 (R) *	0.15 ± 0.03 (H) vs. 0.17 ± 0.04 (R) *
**Reconstructed Motor Movement Data**		
Frequency band	Spectral correlation (Partition vs. Random)	nRMSE (Partition vs. Random)
Delta (1–4 Hz)	0.83 ± 0.05 (P) vs. 0.80 (R) ± 0.05 *	0.12 ± 0.02 (P) vs. 0.14 ± 0.03 (R) *
Theta (4–8 Hz)	0.84 ± 0.04 (P) vs. 0.81 (R) ± 0.06 *	0.09 ± 0.02 (P) vs. 0.11 ± 0.03 (R) *
Alpha (8–13 Hz)	0.93 ± 0.03 (P) vs. 0.86 (R) ± 0.05 *	0.07 ± 0.01 (P) vs. 0.10 ± 0.02 (R) *
Beta (13–30 Hz)	0.87 ± 0.04 (P) vs. 0.82 (R) ± 0.06 *	0.08 ± 0.01 (P) vs. 0.11 ± 0.03 (R) *
Gamma (30–50 Hz)	0.77 ± 0.06 (P) vs. 0.71 (R) ± 0.05 *	0.13 ± 0.02 (P) vs. 0.15 ± 0.03 (R) *

* *p* < 0.05.

**Table 5:** The average and standard deviation of the reconstruction errors (RMSE) across all samples when reconstructing 1 to 10 channels out of 29.

**Resting State:**				
Missing channels	Hemispheric symmetry rotation	Partition-based	Order-based	Random
1	0.058 ± 0.003	0.058 ± 0.003	**0.057 ± 0.003**	0.058 ± 0.003
2	0.061 ± 0.003	0.061 ± 0.003	**0.061 ± 0.003**	0.063 ± 0.004
3	**0.064 ± 0.003**	0.064 ± 0.003	0.064 ± 0.004	0.067 ± 0.004
4	**0.068 ± 0.004**	0.069 ± 0.004	0.068 ± 0.004	0.071 ± 0.005
5	**0.072 ± 0.004**	0.073 ± 0.004	0.072 ± 0.005	0.073 ± 0.007
6	0.076 ± 0.005	0.077 ± 0.006	0.076 ± 0.007	**0.074 ± 0.005**
7	**0.075 ± 0.004**	0.075 ± 0.004	0.075 ± 0.005	0.078 ± 0.007
8	**0.078 ± 0.004**	0.079 ± 0.005	0.078 ± 0.005	0.082 ± 0.008
9	**0.082 ± 0.008**	0.082 ± 0.009	0.083 ± 0.011	0.084 ± 0.009
10	0.084 ± 0.009	0.085 ± 0.009	0.083 ± 0.008	**0.083 ± 0.009**
**Motor Movement:**				
Missing channels	Hemispheric symmetry rotation	Partition-based	Order-based	Random
1	0.048 ± 0.002	0.048 ± 0.002	**0.047 ± 0.002**	0.048 ± 0.002
2	0.05 ± 0.002	0.05 ± 0.002	**0.05 ± 0.002**	0.051 ± 0.002
3	0.053 ± 0.002	**0.052 ± 0.002**	0.053 ± 0.001	0.054 ± 0.003
4	0.055 ± 0.003	**0.055 ± 0.003**	0.056 ± 0.003	0.056 ± 0.004
5	0.057 ± 0.002	**0.056 ± 0.002**	0.057 ± 0.002	0.06 ± 0.005
6	0.059 ± 0.003	**0.058 ± 0.003**	0.059 ± 0.003	0.064 ± 0.005
7	0.061 ± 0.003	**0.061 ± 0.003**	0.063 ± 0.004	0.069 ± 0.005
8	0.064 ± 0.004	**0.063 ± 0.003**	0.065 ± 0.004	0.067 ± 0.007
9	0.064 ± 0.003	**0.064 ± 0.003**	0.067 ± 0.004	0.067 ± 0.005
10	**0.068 ± 0.006**	0.068 ± 0.007	0.07 ± 0.008	0.07 ± 0.005
**Motor Imagery:**				
Missing channels	Hemispheric symmetry rotation	Partition-based	Order-based	Random
1	0.05 ± 0.002	0.051 ± 0.002	**0.05 ± 0.002**	0.051 ± 0.002
2	0.054 ± 0.002	**0.054 ± 0.002**	0.054 ± 0.002	0.056 ± 0.003
3	0.057 ± 0.002	**0.056 ± 0.002**	0.057 ± 0.002	0.059 ± 0.003
4	0.06 ± 0.003	**0.059 ± 0.002**	0.061 ± 0.003	0.062 ± 0.004
5	0.062 ± 0.002	**0.062 ± 0.002**	0.064 ± 0.002	0.067 ± 0.009
6	0.064 ± 0.003	**0.063 ± 0.003**	0.065 ± 0.003	0.07 ± 0.005
7	0.07 ± 0.009	**0.068 ± 0.007**	0.072 ± 0.009	0.068 ± 0.005
8	0.071 ± 0.004	**0.069 ± 0.004**	0.074 ± 0.006	0.072 ± 0.005
9	0.069 ± 0.004	**0.068 ± 0.003**	0.071 ± 0.004	0.078 ± 0.008
10	0.074 ± 0.011	**0.072 ± 0.009**	0.075 ± 0.011	0.079 ± 0.018

The best performance among the four gating mechanisms is highlighted in bold.

**Table 6:** Wilcoxon signed-rank test between the random sequence and hemispheric symmetry rotation gate control mechanisms, and between the partition-based and order-based gate control mechanisms.

	19-Channel			29-Channel		
	EO	MI	MM	EO	MI	MM
Hemispheric symmetry rotation vs. Random	0.001	0.002	0.008	0.005	0.000	0.000
Partition-based vs. Random	0.026	0.007	0.006	0.047	0.000	0.000
Order-based vs. Random	0.026	0.228	0.278	0.003	0.003	0.000

**Table 7:** Subject identification results with 0, 1, 5, and 10 reconstructed channels out of 19 and 29 total channels.

**Motor Movement:**				
#Missing channels/#Total channels	Gate control mechanisms			
	Hemispheric symmetry rotation	Partition-based	Order-based	Random
0/19	87.77			
1/19	86.06	**86.35**	85.46	85.04
5/19	85.53	**85.92**	85.17	84.19
10/19	81.96	**82.34**	81.03	80.72
0/29	92.97			
1/29	92.04	**92.17**	91.35	90.85
5/29	89.73	**90.21**	88.92	89.81
10/29	88.21	**88.35**	87.77	86.48
**Motor Imagery:**				
#Missing channels/#Total channels	Gate control mechanisms			
	Hemispheric symmetry rotation	Partition-based	Order-based	Random
0/19	87.16			
1/19	86.33	**86.48**	85.68	85.43
5/19	84.65	**85.07**	84.8	84.07
10/19	80.57	**81.18**	79.88	80.09
0/29	95.72			
1/29	94.86	**95.08**	92.08	92.41
5/29	90.91	**92.64**	89.15	89.83
10/29	**91.59**	91.29	90.22	85.78

The best performance among the four gating mechanisms is highlighted in bold.

## Data Availability

The original data presented in the study are openly available in PysioNet EEG Motor Movement Imagery Dataset, DOI: 10.13026/C28G6P. The code is openly available in Weighted Gate Layer Autoencoders, Code Ocean Capsule: 4751126.
